# Sputtered Electrolyte-Gated Transistor with Temperature-Modulated Synaptic Plasticity Behaviors

**DOI:** 10.1021/acsaelm.2c00395

**Published:** 2022-05-18

**Authors:** Yang Ming Fu, Hu Li, Tianye Wei, Long Huang, Faricha Hidayati, Aimin Song

**Affiliations:** † Department of Electrical and Electronic Engineering, The University of Manchester, Manchester M13 9PL, U.K.; ‡ Shandong Technology Center of Nanodevices and Integration, State Key Laboratory of Crystal Materials, School of Microelectronics, Shandong University, Jinan 250101, China

**Keywords:** electrolyte gated transistor, synaptic transistor, temperature dependence, synaptic memory, pattern memory

## Abstract

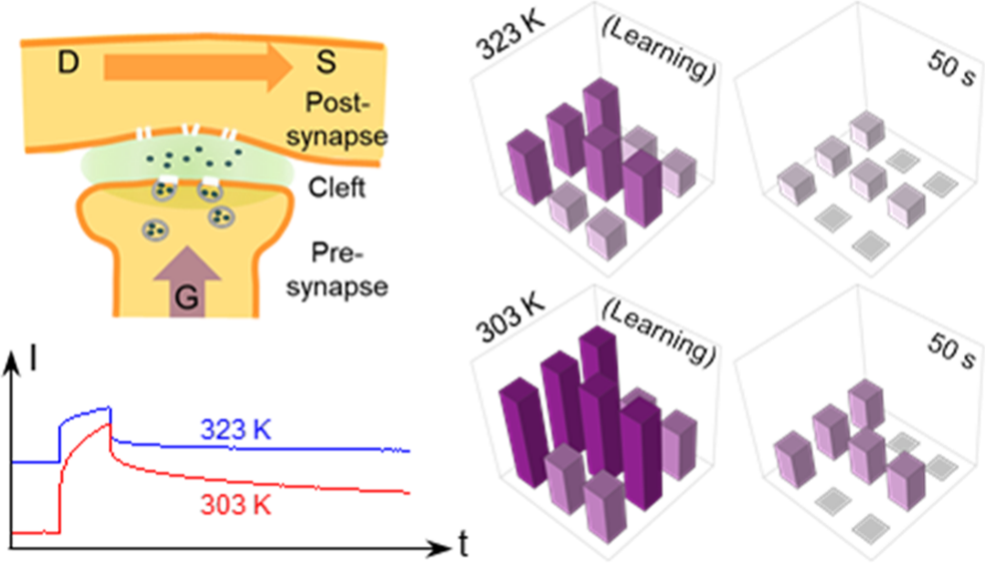

Temperature has always been considered as an essential factor for almost all kinds of semiconductor-based electronic components. In this work, temperature-dependent synaptic plasticity behaviors, which are mimicked by the indium–gallium–zinc oxide thin-film transistors gated with sputtered SiO_2_ electrolytes, have been studied. With the temperature increasing from 303 to 323 K, the electrolyte capacitance decreases from 0.42 to 0.11 μF cm^–2^. The mobility increases from 1.4 to 3.7 cm^2^ V^–1^ s^–1^, and the threshold voltage negatively shifts from −0.23 to −0.51 V. Synaptic behaviors under both a single pulse and multiple pulses are employed to study the temperature dependence. With the temperature increasing from 303 to 323 K, the post-synaptic current (PSC) at the resting state increases from 1.8 to 7.3 μA. Under a single gate pulse of 1 V and 1 s, the PSC signal altitude and the PSC retention time decrease from 2.0 to 0.7 μA and 5.1 × 10^2^ to 2.5 ms, respectively. A physical model based on the electric field-induced ion drifting, ionic–electronic coupling, and gradient-coordinated ion diffusion is proposed to understand these temperature-dependent synaptic behaviors. Based on the experimental data on individual transistors, temperature-modulated pattern learning and memorizing behaviors are conceptually demonstrated. The in-depth investigation of the temperature dependence helps pave the way for further electrolyte-gated transistor-based neuromorphic applications.

## 1 Introduction

Iontronics is an emerging interdisciplinary concept that exploits the ion-controlled or ion-mediated electronic behaviors rather than the ionic or electronic behaviors alone.^1^ In biosystems, the majority of signals are transmitted via ions or molecules rather than electrons.^2^ Hardware implementation of iontronics, that is, iontronic devices, which bridge biology and electronic technology,^3^ has enriched advancements in bioinspired applications,^4^ including bioelectric interfaces^5−9^ and synaptic electronics.^10−14^ An electrolyte-gated transistor (EGT) is one typical class of iontronic devices.^15^ EGTs employ electrolytes as the gate dielectric, enabling ion conduction but electron insulation. Under an applied gate voltage pulse, either ionic–electronic coupling or ionic doping occurs at the electrolyte–semiconductor interface, resulting in an effective modulation of the channel conductance with a relatively long ion-transport relaxation process. The unique ion-mediated modulation makes the EGT ideal for mimicking neural components, where signals are transmitted via neurotransmitter-mediated ionic fluxes.^16−19^

In the last decade, various electrolyte-based EGTs have been developed for neuromorphic applications,^20−23^ such as spin-/drop-coated ion−gel or ion−liquids electrolytes,^24−29^ solution-processed solid electrolytes,^30−34^ plasma-enhanced chemical-vapor-deposition deposited oxide electrolytes,^35−37^ and atomic-layer-deposition deposited oxide electrolytes.^38,39^ Most recently, sputtering was proved to be a cost-effective and, most importantly, industry-compatible way to deposit solid-state oxide electrolytes.^40−43^ Meanwhile, amorphous oxide semiconductors, such as indium–gallium–zinc oxide (IGZO), can also be deposited by sputtering, and they have already been used to commercially manufacture thin-film transistors (TFTs) for display applications.^44,45^ Therefore, amorphous oxide based EGTs gated with a sputtered electrolyte are desirable to achieve low-cost, large-area circuits for potential practical neuromorphic system applications.

Despite the fact that various types of synaptic or neural behaviors and functions have been demonstrated on the amorphous oxide based EGTs,^46,47^ the in-depth understanding of the device operation is still quite limited. For instance, Guo et al. studied the humidity dependence of synaptic behaviors on the indium–tin-oxide transistors gated by plasma-enhanced chemical-vapor-deposition deposited SiO_2_ electrolytes.^48,49^ Enhanced synaptic facilitation was observed at higher relative humidity due to the strengthened proton gating effect. Godo et al. studied the temperature dependence of amorphous IGZO TFTs.^50^ With increasing temperature from 298 to 453 K, the threshold voltage negatively shifted from ∼1 to ∼−4 V. Li et al. studied the temperature influence of a floating gate synaptic transistor based on inorganic perovskite quantum dots. Temperature-modulated synaptic plasticity and accelerated learning behaviors were demonstrated.^51^ Zhu et al. studied the temperature influence of chitosan electrolyte-gated IGZO synaptic transistors. Temperature-induced synaptic functions and spiking logic switching behaviors were demonstrated.^52^ However, to the best of our knowledge, the temperature dependence of an inorganic electrolyte-based EGT with an amorphous oxide semiconductor has not been reported. The temperature has always been a non-negligible factor for almost all semiconductor devices. Similar to the importance of temperature for Si-based central processing units where thermal throttling must be taken into consideration, the temperature dependence of EGT-based synaptic transistors also needs to be studied for further applications in neuromorphic systems.

In our previous work, tunable short-term and potential long-term synaptic behaviors have been demonstrated on sputtered SiO_2_ electrolyte-gated IGZO TFTs.^53^ Herein, we study the temperature dependence of the IGZO EGTs with sputtered SiO_2_ electrolytes. For the transistor performances, the mobility increases, while the threshold voltage negatively shifts with increasing temperature. For the synaptic behaviors, the post-synaptic current (PSC) increases, but the PSC peak altitude and the PSC retention time decrease with increasing temperature. This work gives an in-depth understanding of the temperature dependence of the electrolyte-gated synaptic transistor.

## 2 Experimental Section

**Figure 1:**
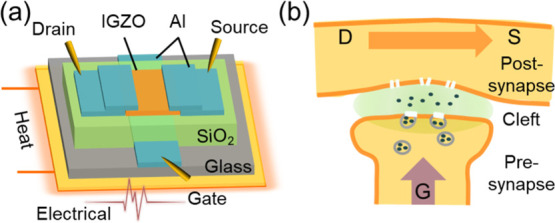
(a) Schematic diagram of the sputtered IGZO synaptic transistor. (b) Simplified schematic of a biosynapse.

Figure 1a shows the schematic diagram of the proposed IGZO synaptic transistor in this work. First, a layer of a patterned Al film was deposited via thermal evaporation, serving as the bottom gate electrode. Then, a 100 nm thick SiO_2_ film was deposited by radio frequency sputtering at 140 W in an Ar atmosphere, serving as the gate dielectric. After that, a 30 nm thick IGZO film was also deposited by sputtering at 90 W, serving as the semiconductor channel. Finally, Al source/drain contacts were deposited via thermal evaporation. All patterns were defined using shadow masks. Meanwhile, sandwiched Al/SiO_2_/Al capacitors were also fabricated with different masks. A Keysight E4980A LCR meter was used to measure capacitor characteristics. An Agilent E5260B semiconductor analyzer was used to measure transistor characteristics and synaptic behaviors. All devices were measured under the dark condition in a Faraday cage chamber.

## 3 Results and Discussion

### 3.1 Synapse Emulation Protocol

Figure 1b shows a simplified schematic of a biosynapse. Here, the stimulus from the pre-synapse neuron causes the release of neurotransmitters to the synapse cleft. Some of these neurotransmitters then bind to receptors on the post-synapse neuron. Many of these receptors contain ion channels capable of passing specific ions (K^+^, Na^+^, etc.) either into or out of the cell, leading to a temporary depolarization or hyperpolarization of the post-synaptic membrane.^54,55^ Neurobiologically, the PSC refers to the ion flow that causes the post-synaptic neuron to be more or less likely to fire an action potential. The synaptic weight refers to the connection strength between the pre- and post-neurons, corresponding to the amount of influence the firing of one neuron has on another. The synaptic weight can be modulated in response to synapse spiking activities.^56^ The neurotransmitter-mediated synaptic weight modulation is very similar to the ion-mediated channel conductance modulation in our sputtered IGZO EGTs. When treating the sputtered IGZO EGT as a synaptic transistor, gate voltage pulses are regarded as pre-synaptic spikes. Channel conductance and channel currents are regarded as the synaptic weight and PSCs, respectively.

### 3.2 Temperature-Modulated Device Performance

**Figure 2:**
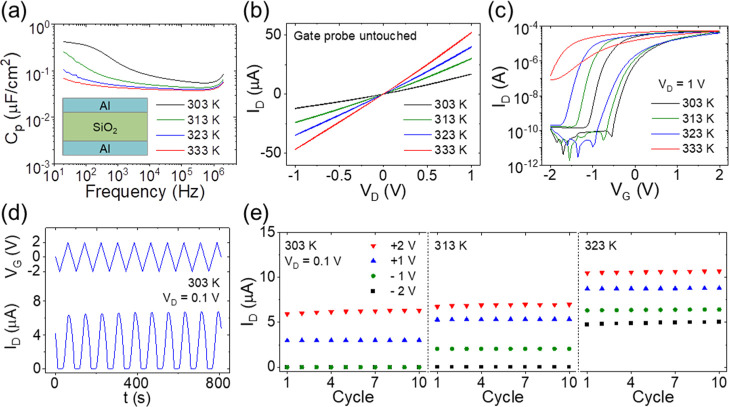
(a) Frequency-dependent specific capacitance of the sputtered SiO_2_ electrolyte at different temperatures. The inset is the schematic of the measured capacitor. (b) Current–voltage curves of the IGZO EGT with the gate probe untouched at different temperatures. (c) Transfer curves of the IGZO EGT at different temperatures. (d) Current response under gate voltage sweep within the ±2 V range at 303 K. (e) ON/OFF currents under voltage sweeps in the range of ±1 and ±2 V at different temperatures.

Figure 2a shows the specific capacitance of the 100 nm SiO_2_ film with the frequency sweeping from 200 kHz to 20 Hz at different temperatures. The inset of Figure 2a shows the schematic of the measured Al/SiO_2_/Al capacitor. At 303 K, the capacitance shows a clear increase with decreasing frequency, indicating the formation of an electric double layer (EDL) at low frequency. A maximum capacitance of 0.42 μF cm^–2^ was obtained at 20 Hz. With increasing temperature, the capacitance exhibited less dependence on the frequency. At 333 K, the capacitance only slightly increased from 0.04 to 0.07 μF cm^–2^ with the frequency decreasing from 100 kHz to 20 Hz, indicating a weakened EDL coupling effect. As ions within porous oxide electrolytes are reported to be most likely protons,^57,58^ the decrease of capacitance may be because of less availability of water molecules at a higher temperature. Figure 2b shows the current–voltage curves of the IGZO EGT at different temperatures with the gate probe untouched. Here, the current implies the intrinsic semiconductor channel conductance without gate modulation. At each temperature, the measured currents exhibited good linearity upon applied voltages, indicating good Ohmic contacts. The channel conductance also increased with increasing temperature. This can be ascribed to extra carriers activated by the thermal energy.

Figure 2c shows the transfer curves of the IGZO EGT at different temperatures. The drain voltage, *V*_D_, was fixed at 1 V. The gate voltage, *V*_G_, was swept from −2 to +2 V and then backward at a rate of 100 mV/s. Clear anticlockwise hysteresis was observed at all temperatures, indicating the occurrence of EDL coupling. At 303 K, the channel current, *I*_D_, increased from 1.4 × 10^–10^ to 5.1 × 10^–5^ A, and the turn-on voltage was about −0.6 V. With increasing temperature, the turn-on voltage negatively shifted to −0.8 and −1.0 V at 313 and 323 K, respectively. At 333 K, the transistor almost lost its transfer characteristic. Table 1 lists the electric parameters extracted from the measured transfer curves (forward sweep). The mobility, μ, is calculated from eq 1 given below
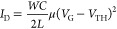
1where *W* and *L* are the channel width and length, respectively. *C* is the electrolyte capacitance. *V*_TH_ is the threshold voltage. With increasing temperature from 303 to 323 K, μ increased from 1.4 to 3.7 cm^2^ V^–1^ s^–1^, while *V*_TH_ negatively shifted from −0.23 to −0.51 V. The current under 0 V gate voltage, *I*_0V_, increased from 0.7 to 2.0 μA. Although the transfer curves varied with different applied voltages (detailed transfer curves are shown in Figure S1), the overall tendency of the negatively shifted transfer curve is very clear. This can be explained from the perspective of the channel and the dielectric. For the channel part, the semiconductor conductance increases with increasing temperature. For the dielectric part, the electrolyte capacitance decreases with increasing temperature, resulting in less effective gate modulation. The fabricated transistors can operate under a higher gate voltage up to 6 V (Figure S2). Figure 2d shows channel currents under gate voltage sweep within the ±2 V range at 303 K. Here, *V*_D_ was fixed at 0.1 V, and *V*_G_ was linearly swept between −2 and +2 V at a rate of 100 mV/s. The current at the highest and lowest voltage is defined as the ON and OFF currents, respectively. Here, the ON currents are around 6.2 μA, and the OFF currents are around 0 μA. Figure 2e shows the ON and OFF currents under voltage sweeps of ±1 and ±2 V at different temperatures. At each temperature, the ON/OFF currents remained stable in the 10 cycles’ sweep. With increasing temperature, both the ON and OFF currents increased. Detailed current responses under voltage sweeps at different temperatures are shown in Figure S2.

**Table 1:** Electric Parameters of the IGZO EGT at Different Temperatures (Forward Sweep)

*T* (K)	*C* (μF cm^–2^)	μ (cm^2^ V^–1^ s^–1^)	*V*_TH_ (V)	*I*_0V_ (μA)
303	0.42	1.4	–0.23	0.7
313	0.26	2.0	–0.35	1.3
323	0.11	3.7	–0.51	2.0
333	0.07			15.6

### 3.3 Emulation of Synaptic Responses and Synaptic Memory Behaviors

**Figure 3:**
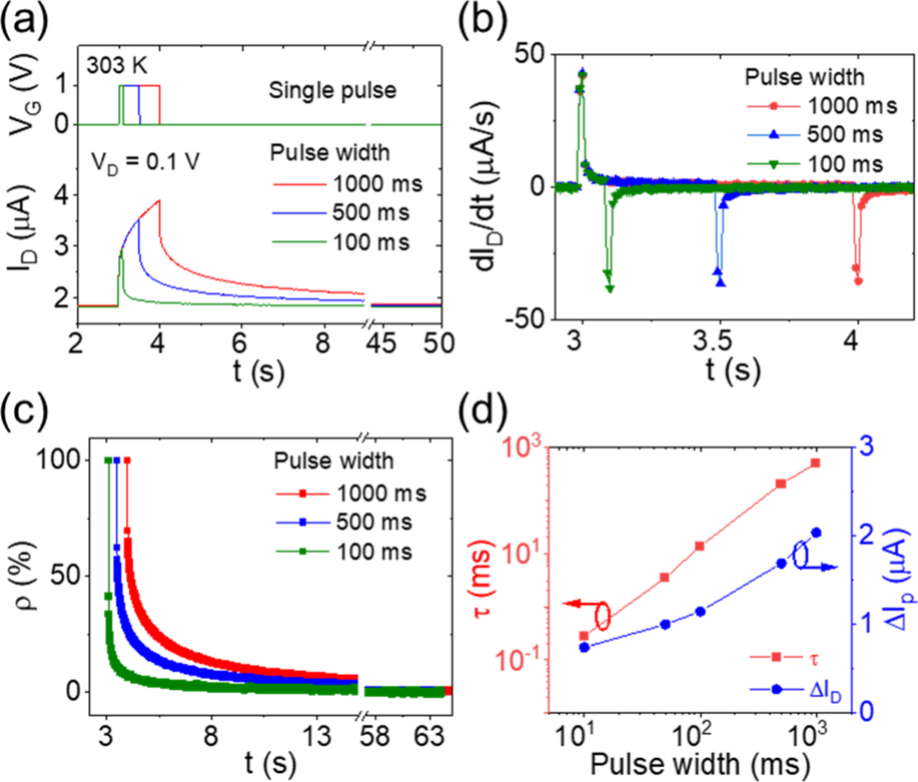
(a) PSC curves and (b) their corresponding derivative curves under a single gate pulse of 1 V height with varied pulse widths (100, 500, 1000 ms) at 303 K. (c) Normalized PSC retention curves. (d) PSC retention times and PSC peak values as a function of the applied pulse width.

Figure 3a shows the PSC curves of the sputtered IGZO synaptic transistor under a single gate voltage pulse of 1 V with varied widths (100, 500, 1000 ms) at 303 K. Here, *V*_D_ was fixed at 0.1 V to read the channel conductance with the grounded source. As shown in Figure 3a, before the pulse, the initial PSC value, *I*_0_, remained stable. During the pulse, the PSC value quickly increased from *I*_0_ to a peak, *I*_p_. After the pulse, the PSC value, *I*_*t*_, gradually decayed back to the initial value. Due to the huge ionic capacitance, the induced electron concentration can be approximately equal to the induced proton concentration.^59^
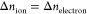
2where *n*_ion_ and *n*_electron_ stand for the ion concentration and electron concentration, respectively. Meanwhile, the induced electron is read by the channel current:
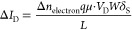
3where δ_S_ and *q* are the depth of the electron concentration layer within the semiconductor and the elementary charge, respectively. Combining eqs 2 and 3, the difference in the channel current should be proportional to the concentration of induced ions
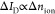
4

Based on the above equations, the sharp increase during the pulse and the gradual decay after the pulse can be explained as follows. Before the pulse arrives, mobile protons are distributed in the equilibrium position within the SiO_2_ electrolyte film, leading to a stable base value. During the pulse period, EDLs are formed at the SiO_2_–IGZO interface by the applied gate electric field, resulting in a sharp increase of the PSC value. After the pulse, the accumulated protons gradually diffuse away to reach an equilibrium. However, the EDL dissipation process is slower than the formation process without the assistance of an external electric field, resulting in gradually decayed PSC curves. Figure 3b shows the corresponding derivatives of the PSC curves. Here, the derivative values were positive during the pulse but negative after the pulse, corresponding to the increase and decay of the PSC value. As the induced ion concentration is proportional to the change in the channel current, the derivative of ion concentration should also be proportional to the derivative of the channel current:
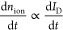
5

In this case, the positive derivative and negative derivative also refer to the accumulation (increase of ion concentration) and dissipation (decrease of ion concentration) of ions, respectively. It was also observed that the first two data points at the beginning of the pulse were much larger than the later data points during the pulse. There might be some possible reasons, such as the inherent field-effect modulation besides the ion-mediated modulation, the built-in electric field formed after the ion migration, and so forth. For similar reasons, the first two data points of the derivative value right after the ending of the pulse were also much lower than the later data points.

Figure 3c shows the normalized PSC retention curves (normalized from the PSC peak value). The PSC retention ratio, ρ, is the ratio between the remaining PSC value, Δ*I*_*t*_ = *I*_*t*_ – *I*_0_, and the PSC peak value, Δ*I*_p_ = *I*_p_ – *I*_0_. It was observed that ρ gradually decays from 100 to 0% within a period after the pulse. The memory retention curves can be fitted by the Ebbinghaus forgetting model:^60,61^
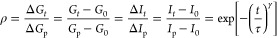
6where ρ stands for the memory retention level, *t* is the time, τ is the memory retention time, and γ is an index between 0 and 1. *G*_*t*_, *G*_p_, and *G*_0_ are the channel conductance at time t, at the end of all gate voltage pulses, and before all the pulses, respectively. Figure 3d shows the PSC peak value and the memory retention time as a function of the applied pulse width. With the pulse width increasing from 10 to 1000 ms, Δ*I*_P_ and τ increased from 0.7 to 2.0 μA and 0.3 to 5.1 × 10^2^ ms, respectively. Detailed fitting curves are shown in Figure S3. The spiking-duration-dependent behaviors can be ascribed to the enhanced EDLs formed with longer pulses. Generally, a longer pulse tend to accumulate an increased number of protons to form the EDLs at the interface, resulting in a greater channel conductance increase and a longer dissipation time.^59^ The sputtered IGZO synaptic transistor also shows spiking-number-dependent behaviors, as shown in Figure S4. Under multiple gate pulses (1 V, 10 ms, 50 Hz), when increasing the pulse number from 1 to 1000, Δ*I*_P_ and τ increased from 0.7 to 2.9 μA and 0.3 to 5.2 × 10^3^ ms, respectively. This can be explained by the temporal coupling effect of the EDL. When multiple pulses are shortly coupled, some of the accumulated protons induced by the previous pulse can remain at the interface when the later pulses come. Then, the next pulse will induce accumulation of more protons at the interface. Therefore, the increased number of pulses tends to accumulate increased number of protons to form enhanced EDLs at the interface, resulting in a greater channel conductance increase and a longer dissipation time.

### 3.4 Temperature-Modulated Synaptic Behaviors under Single Pulses

**Figure 4:**
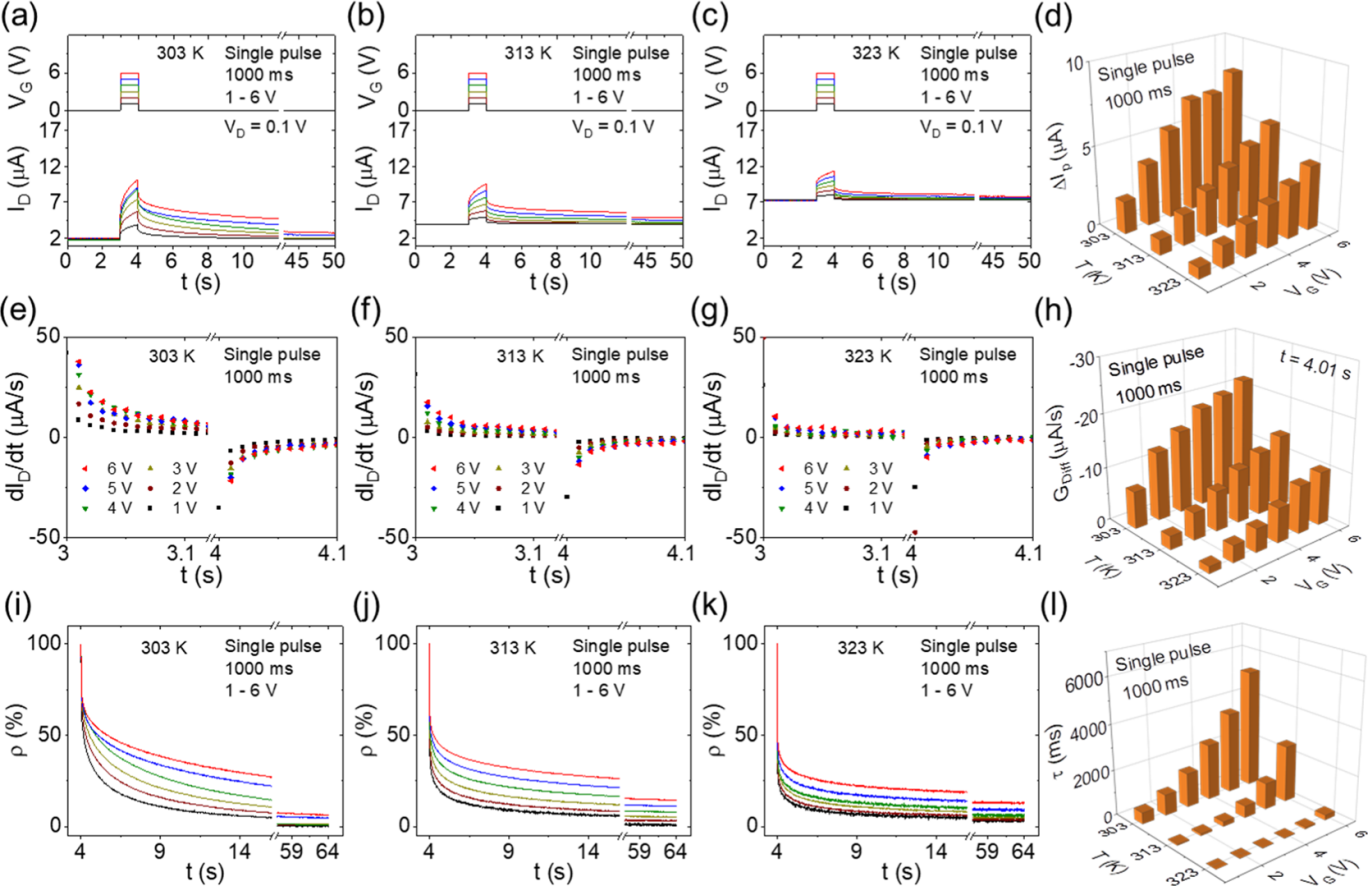
Temperature dependence of synaptic behavior under a single gate pulse. (a–c) PSC curves, (e–g) corresponding derivative curves, and (i–k) normalized PSC retention curves under a single pulse of 1000 ms with varied pulse voltages (1–6 V) at (a,e,i) 303 K, (b,f,j) 313 K, and (c,g,k) 323 K. (d) Normalized PSC peak values, (h) derivative values at 4.01 s (right after the pulse), and (l) PSC retention times as a function of temperature and applied pulse voltage.

Figure 4 shows the temperature dependence of synaptic behavior under a single gate pulse, where Figure 4a–c shows PSC curves at 303, 313, and 323 K, respectively. *V*_D_ was fixed at 0.1 V. *V*_G_ was applied with pulses of 1000 ms with increasing pulse height from 1 to 6 V at a step of 1 V. Each gate voltage pulse caused a PSC peak, and the PSC peak increased with increasing pulse voltage. The initial PSC value (*I*_0_) increased from 1.8 to 7.3 μA with increasing temperature from 303 to 323 K. Figure 4d shows the PSC peak values (Δ*I*_P_) as a function of the temperature and the pulse voltage. It was observed that Δ*I*_P_ decreased with increasing temperature. At 303 K, Δ*I*_P_ increased from 2.0 to 8.0 μA with the pulse voltage increasing from 1 to 6 V. However, at 323 K, Δ*I*_P_ increased from 0.7 to 4.0 μA. Figure 4e−g shows the corresponding derivatives of the PSC curves at 303, 313, and 323 K, respectively. At each temperature, the derivatives are positive during pulses and negative after pulses, relating to proton accumulation and diffusion periods, respectively. Generally, the positive derivatives rise with increasing pulse voltage, while the negative derivatives tend to do the opposite. This can be explained by the faster accumulation of protons under a stronger electric field. A stronger electric field also caused an increase in the number of accumulated ions, which have a larger concentration gradient and lead to faster diffusion. Figure 4h shows the derivative values at *t* = 4.01 s (the first data point in the diffusion period), *G*_diff_, as a function of the temperature and the pulse voltage. Here, the value of *G*_diff_, although negative, increased with increasing temperature. At 303 K, *G*_diff_ negatively shifted from −7.0 to −21.8 μA/s with increasing pulse voltage from 1 to 6 V. However, at 323 K, *G*_diff_ negatively shifted from −1.3 to −10.0 μA/s. The derivative value at *t* = 3.01 s (the first data point in the drift period), *G*_drif_, is shown in Figure S4b. Figure 4i–k shows corresponding normalized PSC retention curves at 303, 313, and 323 K, respectively. The PSC retention ratio decreased within 60 s. It was observed that the PSC retention curve decays faster at a higher temperature. At each temperature, ρ increased with increasing pulse voltage. Figure 4l shows the extracted memory retention times as a function of the temperature and the pulse voltage. At 303 K, τ increased from 5.1 × 10^2^ to 5.2 × 10^3^ ms with increasing pulse voltage from 1 to 6 V. However, at 323 K, τ increased from 2.5 to 2.3 × 10^2^ ms. The sharp decrease exhibits strong dependence on temperature of synaptic behavior under a single pulse.

### 3.5 Temperature-Modulated Synaptic Behaviors under Multiple Pulses

**Figure 5:**
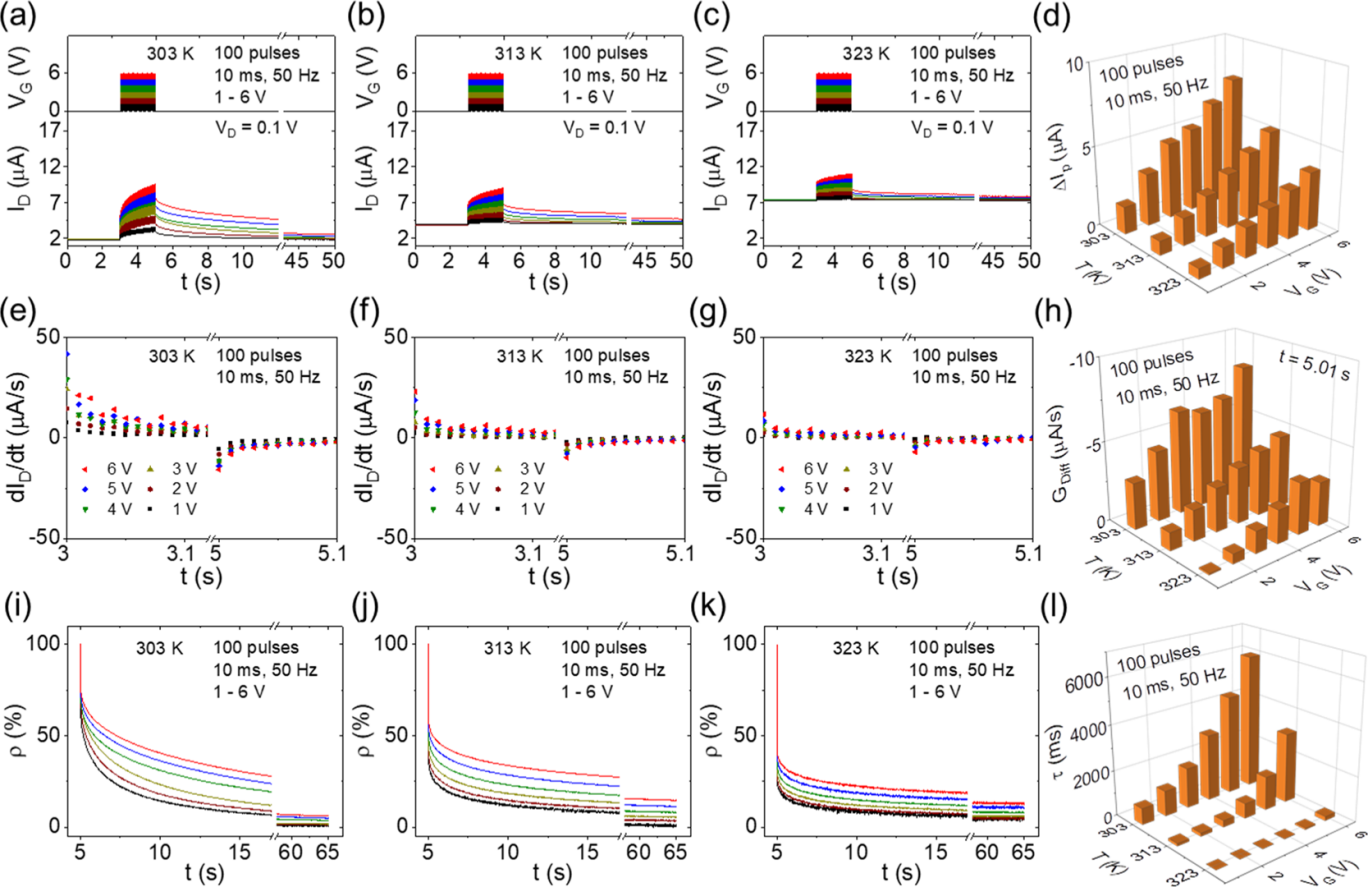
Temperature dependence of synaptic behavior under 100 gate pulses. (a–c) PSC curves, (e–g) corresponding derivative curves, and (i–k) normalized PSC retention curves under 100 pulses (pulse width 10 ms; pulse frequency 50 Hz) with varied pulse voltages (1–6 V) at (a,e,i) 303 K, (b,f,j) 313 K, and (c,g,k) 323 K. (d) Normalized PSC peak values, (h) derivative values at 5.01 s (right after the last pulse), and (l) PSC retention times as a function of temperature and applied pulse voltage.

Figure 5 shows the temperature dependence of synaptic behavior under 100 pulses, where Figure 5a–c shows PSC curves at 303, 313, and 323 K, respectively. *V*_D_ was fixed at 0.1 V. *V*_G_ was applied at 100 pulses (pulse width 10 ms; pulse frequency 50 Hz) with increasing pulse height from 1 to 6 V. The initial PSC value also increased with increasing temperature. At each temperature, the PSC peak increased with increasing pulse voltage. Figure 5d shows the PSC peak values (Δ*I*_P_) as a function of the temperature and the pulse voltage. It was observed that Δ*I*_P_ decreased with increasing temperature. At 303 K, Δ*I*_P_ increased from 1.7 to 7.7 μA with increasing pulse voltage from 1 to 6 V. While at 323 K, Δ*I*_P_ increased from 0.6 to 3.6 μA. Figure 5e–g shows corresponding derivatives of the PSC curves at 303, 313, and 323 K, respectively. Here, the positive derivatives increased with increasing pulse voltage while the negative derivatives increased (negatively) with increasing pulse voltage. Figure 5h shows the derivative values at *t* = 5.01 s (the first data point in the diffusion period) as a function of the temperature and the pulse voltage. Here, *G*_diff_ also increased (negatively) with increasing temperature. At 303 K, *G*_diff_ negatively shifted from −2.9 to −8.1 μA/s with increasing pulse voltage from 1 to 6 V. While at 323 K, *G*_diff_ negatively shifted from −0.1 to −2.8 μA/s. The derivative value at *t* = 3.01 s (the first data point in the drift period) is shown in Figure S5b. Figure 5i–k shows the corresponding normalized PSC retention curves at 303, 313, and 323 K, respectively. Here ρ decreased within 60 s. At each temperature, ρ increased with increasing pulse voltage. It was also observed that the PSC retention curve decays faster at a higher temperature. Figure 5l shows τ as a function of both the temperature and the pulse voltage. At 303 K, τ increased from 7.2 × 10^2^ to 5.9 × 10^3^ ms with the pulse voltage increasing from 1 to 6 V. However, at 323 K, τ increased from 1.6 to 1.9 × 10^2^ ms. The sharp decrease also exhibits strong dependence on temperature of synaptic behavior under multiple pulses.

### 3.6 Working Mechanism

**Figure 6:**
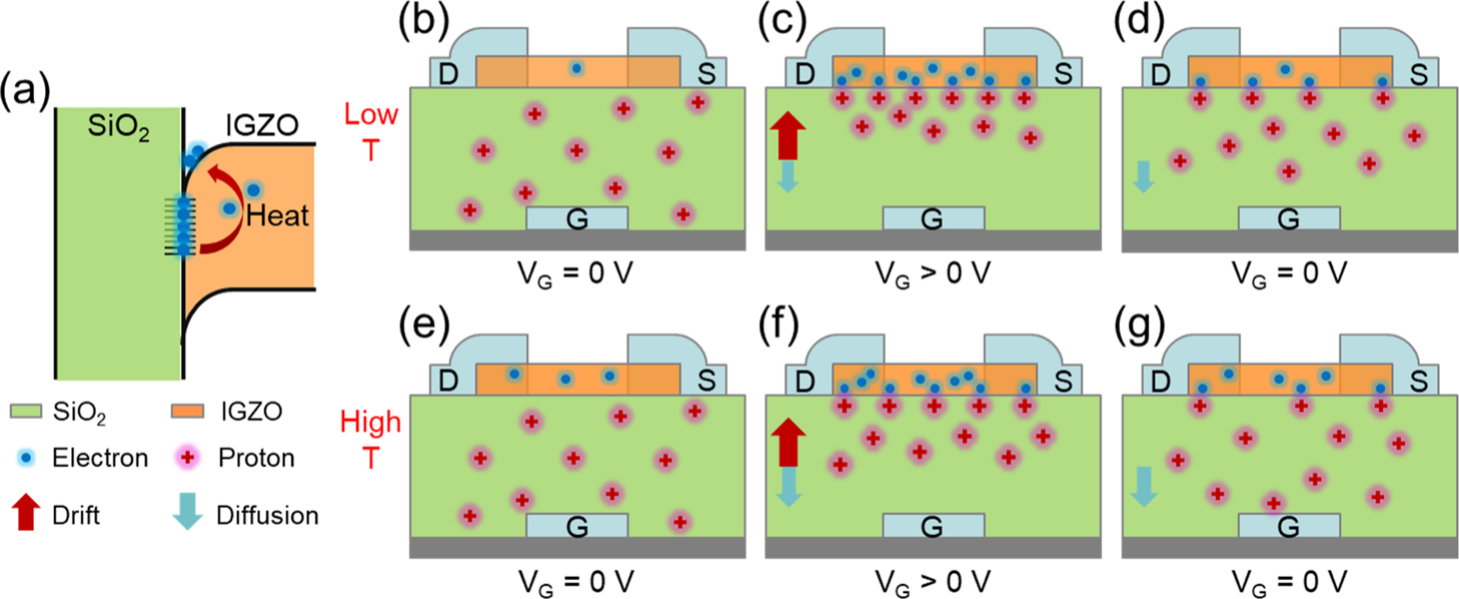
(a) Energy band gap diagram of the IGZO/SiO_2_ interface under heating. Schematic illustration of the transistor working mechanism at a (b–d) low temperature and (e–g) high temperature.

Figure 6 illustrates the underlying working principle of the temperature-dependent synaptic behaviors. Figure 6a shows the energy band gap diagram of the IGZO/SiO_2_ interface under heating. At room temperature, some of the electrons from the IGZO semiconductor channel can be trapped at the interface. With increasing temperature, the trapped electrons can be released, and more intrinsic electrons are also induced by the activation of the thermal energy, resulting in an increased channel conductance.^50^Figure 6a–c and Figure 6e,f illustrate the working mechanisms of the synaptic behavior at a low temperature and a high temperature, respectively. In both cases, before applying the gate voltage pulses (*V*_G_ = 0 V), protons distribute in an equilibrium state, resulting in stable PSC initial values. However, the intrinsic channel conductance increases with increasing temperature. Therefore, *I*_0_ increases with increasing temperature. During the positive gate voltage pulse (*V*_G_ > 0 V), mobile protons migrate to and accumulate at the IGZO–SiO_2_ interface. In addition to the electric field-induced drift, there is also a gradient-induced diffusion for the protons, and such a gradient-induced diffusion effect is enhanced with increasing temperature.^62,63^ This causes the protons to distribute closer to the IGZO–SiO_2_ interface and hence a larger increase in the channel current at a lower temperature,^59^ which means that the PSC peak value (Δ*I*_p_) is higher at lower temperatures. After the pulses (*V*_G_ = 0 V), the applied electric field is withdrawn, and the accumulated protons gradually diffuse back to reach an equilibrium state. Here, the protons at a high temperature diffuse much faster than those at a low temperature, resulting in faster decrease in the PSC curve,^12,51^ which means that the memory retention time (τ) decreases with increasing temperature.

### 3.7 Temperature-Modulated Pattern Learning and Memorizing Behavior

**Figure 7:**
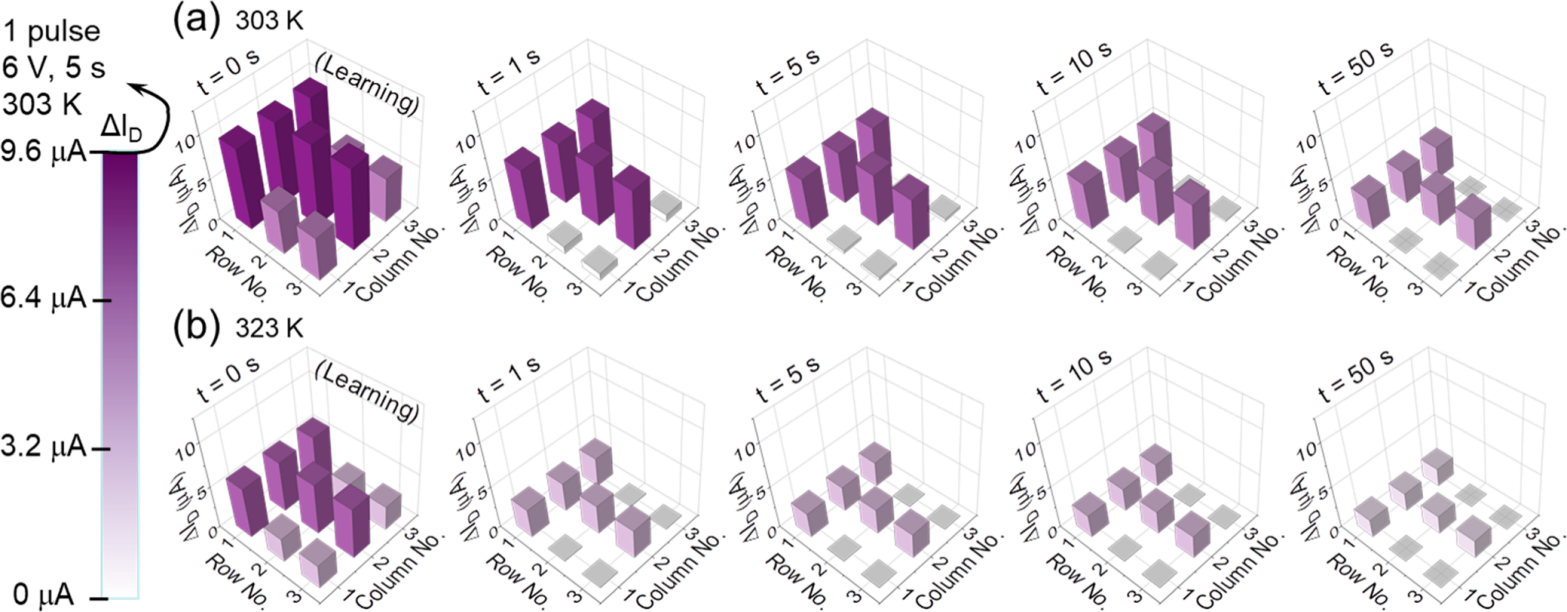
Pattern learning and memorizing behaviors at (a) 303 and (b) 323 K. The 3 × 3 patterns show the conductance value at *t* = 0 s (right after learning), at *t* = 1 s, at *t* = 5 s, at *t* = 10 s, and at *t* = 50 s. The conductance is represented in colors (from pure white to pure purple), and the value is normalized by the PSC peak value under a single pulse of 6 V and 5000 ms at 303 K.

Based on the measured data on individual transistors, a 3 × 3 array of our synaptic transistors was conceptually configured to demonstrate the temperature-modulated pattern learning and memorizing behaviors. As shown in Figure 7, the 3 × 3 pattern (each related to a synaptic transistor) shows the conductance represented in color at different time points (*t* = 0, *t* = 1, *t* = 5, *t* = 10, and *t* = 50 s). Figure 7a,b is taken at 303 and 323 K, respectively. As shown in the index bar, the conductance value is normalized by the PSC peak value (9.6 μA) induced by a single pulse of 6 V and 5000 ms at 303 K. The color is altered from pure white (0%) to pure purple (100%). The writing scheme is shown in Figure S7a. At *t* = 0 s, a “*T*” pattern is written into the synaptic array by an effective signal pulse of 6 V and 5000 ms. Meanwhile, other elements are written by a noise signal pulse of 6 V and 50 ms. This can be regarded as the learning or memory-forming process. Here, the “*T*” pattern is represented in pure purple at 303 K (9.6 μA) but in light purple at 323 K (5.9 μA). The noise PSC signal at 303 K (5.2 μA) is also higher than that at 323 K (2.8 μA). This is because a higher temperature leads to a lower PSC signal altitude. At both temperatures, the purple pattern gradually fades away with time as the memory gradually fades. Within 1 s, both noise PSC signals turn to be less than 1 μA which is negligible for the “*T*” pattern, indicating the effectiveness of the pattern memory. At 303 K, the purple pattern lasts much longer. After 50 s, the “*T*” pattern signals are still beyond 3 μA, which is very well readable. At 323 K, the purple pattern fades relatively faster. After 5 s, the “*T*” pattern signals turn below 3 μA. Here, the high temperature exhibits a negative effect on the pattern memory behavior. Figures S6 and S7b show the temperature-dependent pattern learning and memory behaviors coded by pulse numbers and the writing scheme, respectively. The higher temperature also exhibits an acceleration effect on the forgetting process.

### 3.8 Temperature-Modulated Energy Consumption and the Signal-to-Noise Ratio

**Figure 8:**
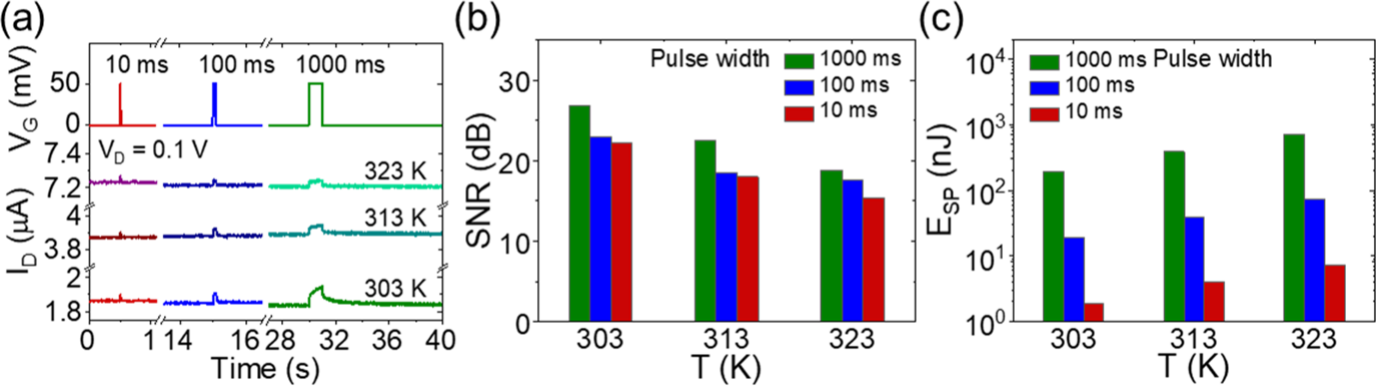
(a) PSC curves under a single gate pulse of 50 mV with varied pulse widths (10, 100, 1000 ms) at different temperatures. (b) SNR and (c) single pulse event energy (*E*_SP_) as a function of temperature.

Besides the PSC peak and the memory retention, we also studied the signal-to-noise ratio (SNR) and the energy consumption at different temperatures. Figure 8a shows PSC curves under a single pulse of 50 mV with varied pulse widths (10, 100, 100 ms) at different temperatures (303, 313, 323 K). It was observed that the signal becomes more distinctive with increasing pulse width but more blurred with increasing temperature. Figure 8b shows the SNR as a function of temperature. At 303 K, the SNR decreased from 27 to 22 dB with the pulse width decreasing from 1000 to 10 ms. Under a single pulse of 10 ms, the SNR decreased from 22 to 15 dB with the temperature increasing from 303 to 323 K. These results can be ascribed to the wider thermal fluctuation at a higher temperature. Figure 8c shows the energy consumed on a single pulse event, *E*_SP_, with different pulse widths (10, 100, 1000 ms) as a function of temperature. It was observed that *E*_SP_ decreased with decreasing pulse width. At 323 K, *E*_SP_ decreased from 724 to 7 nJ with decreasing pulse width from 1000 to 10 ms. Under a 10 ms pulse, *E*_SP_ increased from 2 to 7 nJ with increasing temperature from 303 to 323 K. This increase is mainly because of the increase of the initial PSC value. Figure S7 shows the power at the resting state, *P*_rest_, as a function of temperature. *P*_rest_ also increased from 185 to 721 nW, which is very similar to *E*_SP_. Although here a single pulse event energy is on the nJ level which is not as good as the previously reported fJ level,^38,64^ here our devices is also in the micrometer scale. Once our device downscaled to nanometer with further optimization, it should be able to be reduced to the sub-pJ level.

## 4 Conclusions

In this work, sputtered SiO_2_ electrolytes were utilized to gate the IGZO TFTs to mimic the synaptic response and memory behaviors. Temperature dependence of both the transistors’ performances and the synaptic behaviors was studied. Our obtained results show that the ion behaviors or ion dynamics within the electrolytes, which are highly sensitive to temperature, play an essential role in the device operation. With increasing temperature, ions diffuse faster, leading to a much shorter synaptic memory time. At a higher temperature, ions also tend to form a dispersed distribution, resulting in a lower PSC signal altitude. An in-depth investigation of the temperature influence helps pave the way for further EGT-based neuromorphic applications.

## Supporting Information Available

Detailed transfer curves; transistor performance under higher gate voltages; detailed current response under gate voltage sweeps; detailed fitting of the PSC retention curves; synaptic behavior under multiple pulses; derivative value at the accumulation state; temperature-dependent pattern learning and memorizing behaviors under multiple pulses; learning schemes of the temperature-dependent pattern learning and memorizing behaviors; and power consumption at the resting state (PDF)
