# Supplementary material for: Sputtered Electrolyte-Gated Transistor with Temperature-Modulated Synaptic Plasticity Behaviors
Source: ACS Appl Electron Mater. 2022 May 18;4(6):2933–42. doi: 10.1021/acsaelm.2c00395 (PMC9245437; doi:10.1021/acsaelm.2c00395)
Supplement: Supplementary file 1 — el2c00395_si_001.pdf [file el2c00395_si_001.pdf]

Supporting Information

Sputtered Electrolyte-Gated Transistor with Temperature-Modulated Synaptic Plasticity Behaviors

Yang Ming Fu[†], Hu Li[‡], Tianye Wei[†], Long Huang[†], Faricha Hidayati[†], Aimin Song[†‡]*

†. Department of Electrical and Electronic Engineering, The University of Manchester, Manchester, M13 9PL, UK

‡. Shandong Technology Center of Nanodevices and Integration, State Key Laboratory of Crystal Materials, School of Microelectronics, Shandong University, Jinan, 250101, China

*Corresponding Author: A.Song@manchester.ac.uk

S1. Detailed transfer curves.

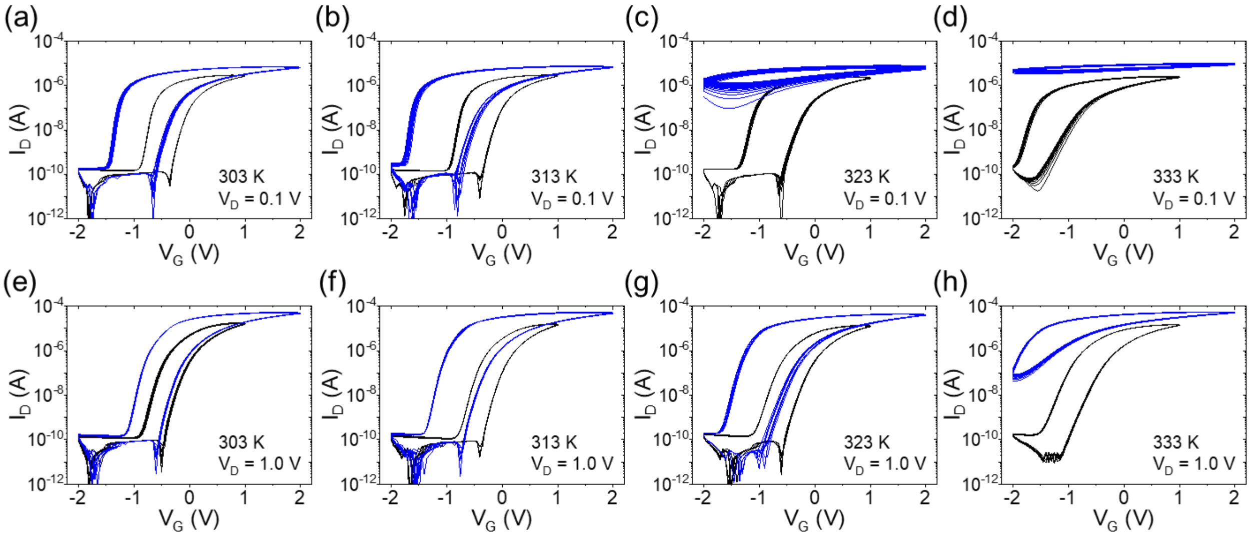

Figure S1. Detailed transfer curves in 10 cycles with different drain voltages at different temperatures. (a, e) 303 K, (b, f) 313 K, (c, g) 323 K, (d, h) 333 K; (a-d) V_D = 0.1 V, (e-h) V_D = 1.0 V.

S2. Transistor performance under higher gate voltages.

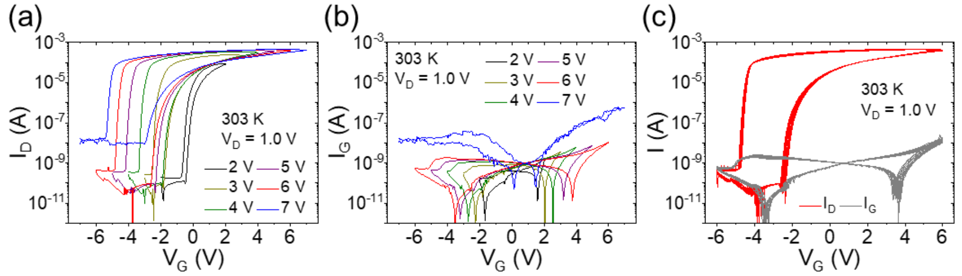

Figure S2. Transistor performance under higher gate voltages. (a) I_D and (b) I_G under gate voltage sweeps from ±2 to ±7 V. (c) I_D and I_G under gate voltage sweeps of ±2 V in 10 cycles.

S3. Detailed current responses under gate voltage sweeps.

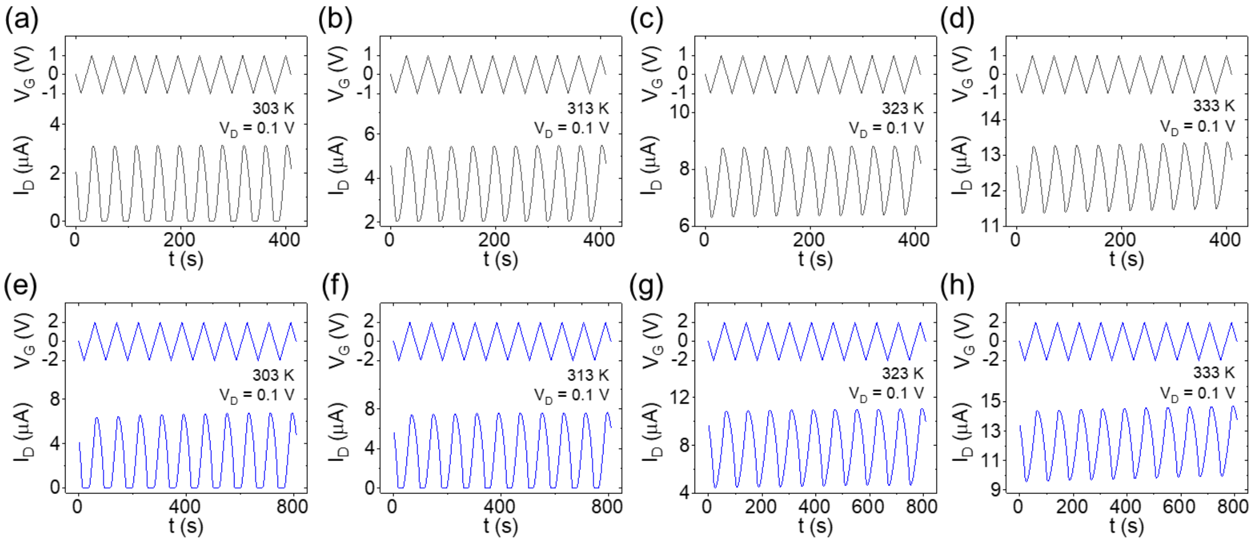

Figure S3. Detailed current response curves under voltage sweeps at different temperatures. (a, e) 303 K, (b, f) 313 K, (c, g) 323 K, (d, h) 333 K; (a-d) V_G range ±1.0 V, (e-h) V_G range ±2.0 V.

S4. Detailed fitting of the PSC retention curves.

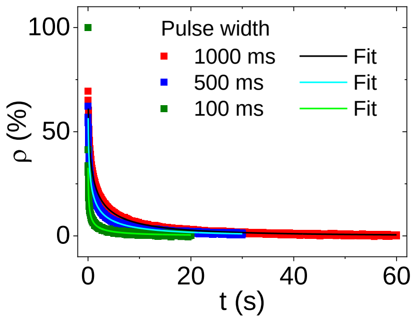

Figure S4. Detailed fitting of the normalized PSC retention curves under a single pulse of 1 V with varied pulse widths (100, 500, 1000 ms).

S5. Synaptic behavior under multiple pulses.

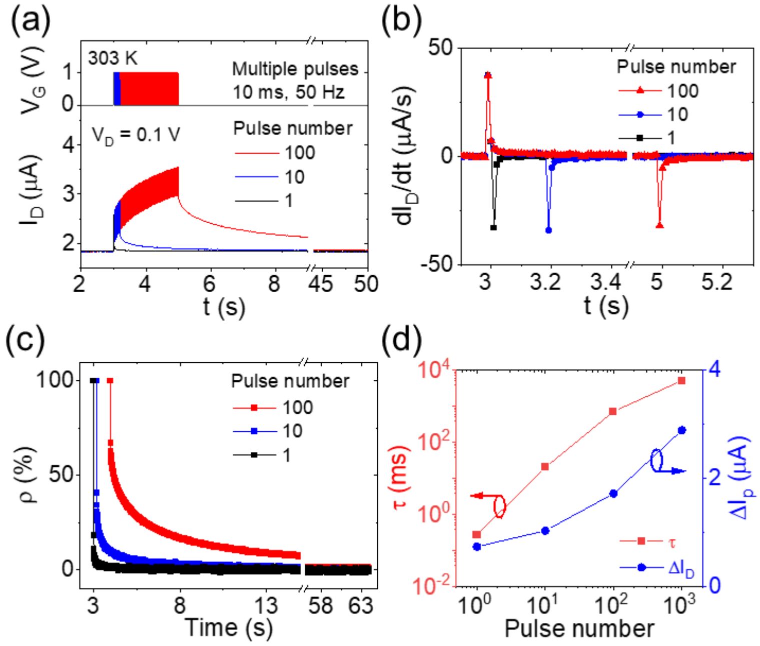

Figure S5. (a) PSC curves and (b) their corresponding derivative curves under multiple gate pulses of 1 V (pulse width 10 ms, pulse frequency 50 Hz) with varied pulse numbers (1, 10, 100) at 303 K. (c) Normalized PSC retention curves. (d) PSC retention time and normalized PSC peak value as a function of applied pulse number.

S6. Derivative value at accumulation state.

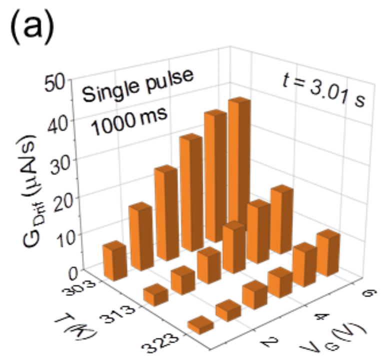

Figure S6. Derivative value at 3.01 s (right after the initiating of the first pulse) of the PSC curves under (a) a single pulse (pulse width 1000 ms) and (b) 100 pulses (pulse width 10 ms, pulse frequency 50 Hz) as a function of the temperature and applied pulse voltage.

S7. Temperature-dependent pattern learning and memorizing behaviors under multiple pulses.

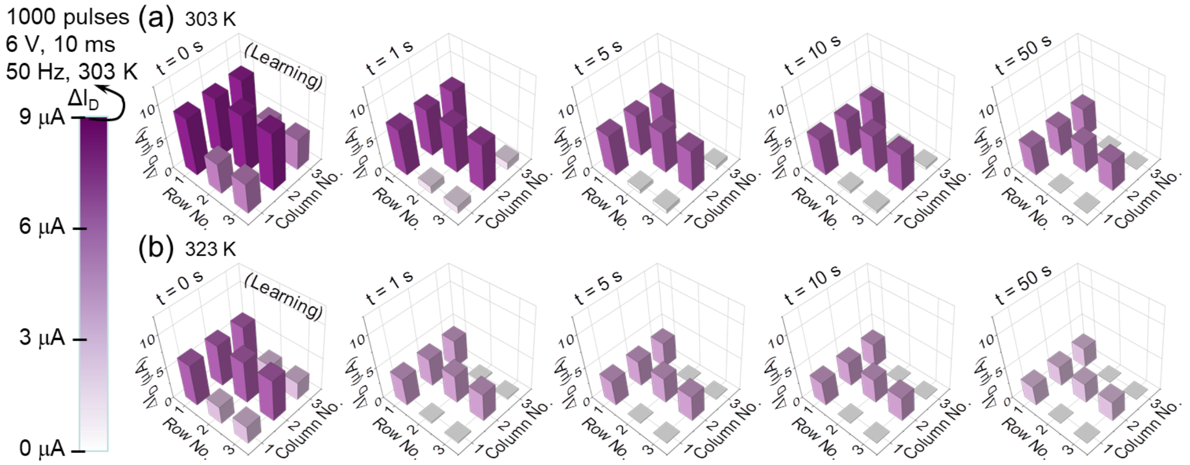

Figure S7. Pattern leaning and memorizing behaviors at (a) 303 K and (b) 323 K. The 3×3 patterns show the conductance value at *t* = 0 s (right after learning), at *t* = 1 s, at *t* = 5 s, at *t* = 10 s, and at *t* = 50 s. The conductance is represented in color (from pure white to pure purple) and the value is normalized by the PSC peak value under 1000 pulses of 6 V, 10 ms and 50 Hz at 303 K.

S8. Leaning schemes of the temperature-dependent pattern learning and memorizing behaviors.

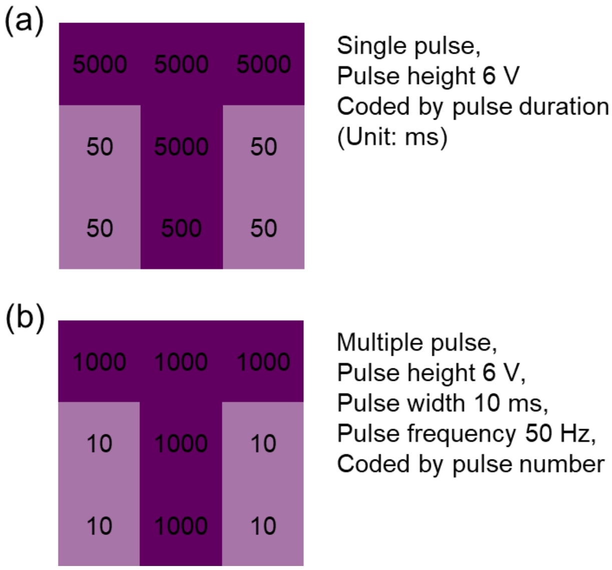

Figure S8. (a) Learning scheme of the spiking duration dependent pattern leaning memory behavior. (b) Learning scheme of the spiking number dependent pattern leaning memory behavior.

S9. Power consumption at resting state.

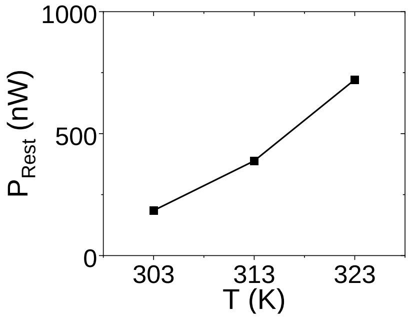

Figure S9. Power consumption at resting state.
